# The Role of the Fox Gene in Breast Cancer Progression

**DOI:** 10.3390/ijms26041415

**Published:** 2025-02-07

**Authors:** Shaoxuan Pei, Dechun Zhang, Zhuohan Li, Jinkai Liu, Ziyi Li, Jianrui Chen, Zhenzhen Xie

**Affiliations:** 1 School of Basic Medical Sciences, Jiangxi Medical College, Nanchang University, Nanchang 330031, China; a15270003301@163.com (S.P.); 15979006926@163.com (D.Z.); 15355186901@163.com (Z.L.); 19970410623@163.com (J.L.); lzy15006333008@163.com (Z.L.); sdchenjianrui@163.com (J.C.); 2 Medical Department, Queen Mary School, Nanchang University, Nanchang 330031, China

**Keywords:** forkhead box (FOX), breast cancer, drug resistance

## Abstract

Forkhead box (FOX) genes are a family of transcription factors that participate in many biological activities, from early embryogenesis to the formation of organs, and from regulation of glucose metabolism to regulation of longevity. Given the extensive influence in the multicellular process, FOX family proteins are responsible for the progression of many types of cancers, especially lung cancer, breast cancer, prostate cancer, and other cancers. Breast cancer is the most common cancer among women, and 2.3 million women were diagnosed in 2020. So, various drugs targeting the FOX signaling pathway have been developed to inhibit breast cancer progression. While the role of the FOX family gene in cancer development has not received enough attention, discovering more potential drugs targeting the FOX signaling pathway is urgently demanded. Here, we review the main members in the FOX gene family and summarize their signaling pathway, including the regulation of the FOX genes and their effects on breast cancer progression. We hope this review will emphasize the understanding of the role of the FOX gene in breast cancer and inspire the discovery of effective anti-breast cancer medicines targeting the FOX gene in the future.

## 1. Introduction

**Figure 1:**
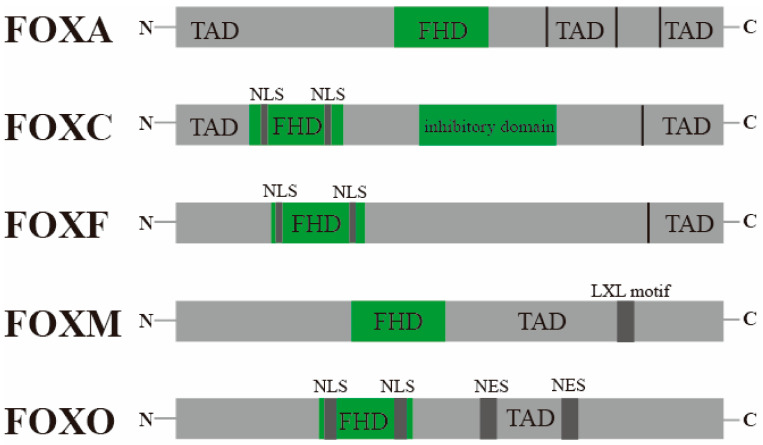
Structure of FOX TFs. FHD: Forkhead box domain; TAD: Transcriptionally Active Domain; NLS: Nuclear Localization Signal; LXL Motif: Leucine-X-Leucine Motif; NES: Nuclear Export Signal.

The Forkhead box (FOX) family is composed oftranscription factors that emerged in unicellular eukaryotes and expanded via multiple duplication events. Overall, the structure of the FOX family always contains a DNA-binding domain (DBD) and a transactivation domain (TAD) [1]. The DBD always consists of 80–100 amino acid residues, including a highly conserved Forkhead box domain (FHD/FKHD) [2]. Classical FHD, with a structure of three N-terminal a-helices (H1–3), three β-sheets (S1–3), and two loops or wings (W1–2) toward its C-terminal region, are generally arranged in order of H1-S1-H2-H3-S2-W1-S3-W2 [3]. The FOX transcription factor specifically recognizes and binds to bases in DNA through some key amino acid residues (e.g.: Asn165 and His169) in its DBD. The helix H3 and the wing W2 seem to be essentially important parts to recognize and bind to the corresponding DNA site [4]. Since the DBD sequence in FOX transcription factors is highly conserved and only binds to DNA containing a specific nucleotide sequence, the binding of both is generally considered to be a highly specific process. The structure and function of transcription activation domains (TADs) in Fox transcription factors exhibit significant variability, lacking a universal consensus sequence or structural motif that defines a TAD in Fox proteins [2] (Figure 1).

The FOX gene family plays significant roles, from early embryogenesis to the formation of organs, and from the regulation of glucose metabolism to the regulation of longevity [5,6,7,8,9,10]. For instance, FOXA proteins are known as “pioneer factors” which can open tightly compacted chromosomes independently. This enables FOXA to regulate the development of the liver, pancreas, lung, prostate, and kidney in adults [11]. Existing studies have shown that FOXA1 can form a regulatory network with estrogen receptor α (ERα) and GATA3 to regulate the occurrence of breast ductal morphology [12] The FOXC factor is crucial in the embryonic and adult stages. It is predominantly found in the embryonic mesoderm and influences numerous physiological processes in adults, including the preservation of podocytes essential for regular kidney operation and vascular modification during tissue injury healing [13,14]. The role of FOXC1 in the development and metastasis of breast cancer has been widely studied. Similarly to other FOX TFs, FOXF mainly plays a role in embryonic development, especially in the development of the digestive system (intestine). The FOXF factor can control epithelial proliferation and development by limiting the Wnt signal in the stroma and maintaining digestive epithelial homeostasis [15,16]. Meanwhile, FOXF also plays a guiding role in the process of embryonic heart separation [17]. FOXM1 is an important cell cycle control factor, which can promote cell division through transcriptionally active factor functions in both the G1/S and G2/M transitions [5]. FOXM1 is physiologically active in the embryo and declines in adult life [18]. So, FOXM1 overexpression in adults usually indicates cancer. FOXO is generally expressed in the liver, adipose tissue, and skeletal muscle [19]. This is closely related to FOXO’s ability to regulate glucose metabolism in those insulin-responsive tissues. Mechanically, FOXO regulates glucose usage by adjusting the sensitivity to insulin. The regulatory role of FOXO in the cell cycle also makes it easy for researchers to associate it with cancer [20]. 

Breast cancer is one of the most prevalent cancers around the world. According to the data from the World Health Organization (WHO), about 2.3 million women were diagnosed with breast cancer, resulting in 685,000 deaths in 2020; one out of eight to ten women will suffer from breast cancer, with a mortality rate of 8% [21]. Based on the receptors that breast cancer cells express, breast cancer is categorized into three main groups: (1) the breast cancer-expressing hormone receptor, which can be further divided into estrogen (ER)-positive breast cancer and progesterone receptor (PR)-positive breast cancer. (2) breast cancer-expressing human epidermal receptor 2 (HER2-positive), and (3) triple-negative breast cancer (TNBC), which does not express ER, PR, or HER. TNBC can be further divided into five groups: basal-like 1/2 (BL1/2), immunomodulatory (IM), mesenchymal (M), mesenchymal stem cell-like (MSL), and the luminal androgen receptor (LAR) [22]. Breast cancer prognoses and treatment strategies depend on the metastasis stage and the category of breast cancer. Early breast cancer without metastasis is potentially curable: resection of the tumor is a viable option for every patient, and primary radiotherapy and systemic therapy such as chemotherapy may be more appropriate for TNBC or HER2-positive breast cancer [21]. ER- and PR-positive breast cancer respond well to endocrine therapy. Regardless of the treatment adopted, early-stage breast cancer patients generally have a favorable prognosis. However, the prognosis for metastatic breast cancer is quite poor. The therapeutic goals are ensuring the quantity of life rather than achieving a cure. So, specific endocrine therapy and chemotherapy are preferred to repress further deterioration.

The molecular mechanisms underlying tumor progression can induce genomic instability, thereby conferring enhanced proliferative capacity on cancer cells, while promoting angiogenesis through the secretion of angiogenic factors, which collectively facilitate tumorigenesis and progression. For instance, the loss-of-function mutation of BRCA1 is a well-known cause of breast cancer cell genome instability [23]. As BRCA1 plays an important role in DNA repair, the inactivation of BRCA1 leads to genome instability and, eventually, tumorigenesis. There are also deregulated pathways that can promote the proliferation of cancer cells. A classic example is the HER2 pathway. The activation of HER2 can subsequently activate three key signaling pathways: the Ras/Raf/MAPK, JAK/Stat, and PI3K/AKT/mTOR pathways. Those pathways are often aberrantly activated in breast cancer, leading to uncontrolled proliferation, survival, invasion, and resistance to therapy [24]. The FOX TFs discussed in this review play a significant role in modulating their activities and are intricately involved in the regulatory mechanisms that drive their functions. Moreover, FOX TFs also participate in the activation of EMT (epithelial–mesenchymal transition), which is crucial for the deterioration and metastasis of breast cancer. Specifically, core EMT factors such as SLUG, TWIST1, and ZEB2 are regulated by the TGF (transforming growth factor)-β/Smad, Wnt/β-Catenin, EGF (epidermal growth factor), Notch, and MAPK signaling pathways. The interactions between these EMT pathways and FOX TFs will be further explored in this review [25].

To date, 44 subfamilies of FOX genes have been documented, ranging from FOXA1 to FOXS1 [5]. Among them, several FOX subfamilies, including FOXA, FOXC, FOXF, FOXM, and FOXO, share many common pathways, such as tumorigenesis, proliferation, epithelial–mesenchymal transition (EMT), and other related pathways. These subfamilies are interconnected through these pathways in breast cancer. In this review, we focus on the regulation pathway and FOX TF’s oncogenic mechanism. On this basis, we discussed the existing methods and drug resistance of FOX TF-based breast cancer treatment.

## 2. Signaling Pathway of FOX

The FOX family members are significantly dysregulated in breast cancer, with their aberrant expression consistently associated with uncontrolled proliferation and poor prognosis. Instead of exerting direct oncogenic effects, these members function as transcription factors, transactivating downstream oncogenes to drive the progression of breast cancer. The detailed mechanisms underlying this process will be elucidated in the following sections (Table 1).

### 2.1. Signaling Pathway of FOXA1

**Figure 2:**
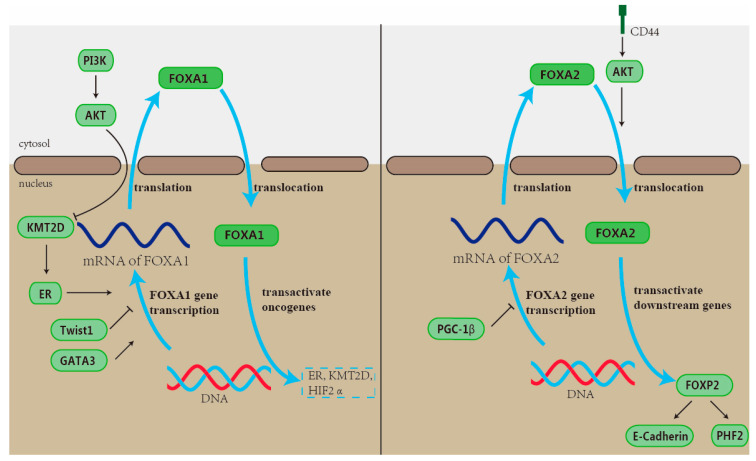
Regulation of FOXA1 and FOXA2, and their oncogene effects. During the progression of breast cancer, dysregulated oncogenes promote or repress the transcription of FOXA1 and FOXA2 genes, regulating their translocation between the nucleus and cytosol to upregulate the effects of FOXA1 and FOXA2 in transactivating downstream oncogenes. FOXA1 and FOXA2 are quite similar in structure, so they may have some overlapping in several pathways. Symbols: ↑ promote, ⊥ inhibit.

FOXA is a family member of Fox transcription factors that play an important role in normal mammary gland development and maintenance. Research has shown that FOXA1 can affect the subtype and prognosis of breast cancer by binding and expressing ER [85]. ER is a nuclear receptor that plays a role in regulating the growth and differentiation of breast cells in normal breast tissue; its expression level is a good prognostic indicator for breast cancer patients [86]. Upon activation of the ER, transcription factors such as FOXA1 are activated. FOXA1 can bind to ER to enhance its transcriptional activity, ultimately promoting breast cell proliferation and differentiation [87] (Figure 2). Mechanistically, FOXA1 is required for nearly all ER-binding events in breast cancer cells [88]. Their expression is positively correlated with breast cancer [86,89]. Research indicated that the transcriptional regulatory network involving estrogen receptors alpha (ESR1), GATA3, and FOXA1 plays a significant role in the development of breast cancer [88,90,91]. GATA3 is a transcription factor that is involved in the luminal differentiation of breast epithelium, and it also plays a role in the maintenance of the luminal cell identity and the suppression of the basal cell phenotype in breast cancer [92]. Vasiliki Theodorou et al. pointed out that GATA3 acts upstream of FOXA1 and affects their binding by regulating ESR1 transcription enhancers [93]. The process results in the enrichment of forkhead and GATA3 DNA motifs within the ESR1-binding regions [94,95]. Then, the overexpression of FOXA1 and GATA3 can enhance ER to a certain extent αReaction [96]. In this case, abnormal activation of the ER in breast cells may lead to abnormal cell proliferation and differentiation, increasing the risk of breast cancer [97]. Similarly, KMT2D is a histone H3 lysine 4 methyltransferase that is necessary for the recruitment and activation of FOXA1, PBX1, and ER [98,99]. Studies by Eneda Toska et al. show that FOXA1 is involved in the PI3K/AKT pathway regulating cancer ER-dependent transcription through the epigenetic regulator KMT2D [27]. AKT interacts with and phosphorylates KMT2D, which in turn reduces its methyltransferase activity and impacts ER function. Concurrently, PI3Ka inhibition enhances KMT2D activity, thereby promoting the recruitment of the FOXA1-PBX1-ER regulatory network and releasing a powerful ER-dependent transcription program, ultimately enhancing the invasiveness of ER-positive breast cancer [27]. For ER-positive breast cancer, the abnormal FOXA1/HIF-2α transcriptional axis has a close relationship with it [28]. A 2019 study suggests that the hypoxia-inducible factor-2α (HIF-2α) is the Super Enhancer (SE) target induced by FOXA1. Instigated by mutation, the upregulation of FOXA1 initiates a comprehensive reprogramming of enhancers across the genome, setting in motion a pretranscriptional program, enabling FOXA1 to employ SE in managing this transcriptional reconfiguration [100,101]. Research has identified that the ability of colony formation, migration, and invasion of breast cancer cells caused by FOXA1 overexpression can be reduced by counteracting HIF-2α [28,102].

Additionally, research conducted by Y Xu and his team revealed that Twist1 can suppress FOXA1 expression, thereby enhancing the invasive and metastatic properties of breast cancer [29]. Twist1 is a principal transcription factor, that triggers embryonic development and cancer cell behaviors such as EMT, cell migration, and invasion, influencing cancer cell activities through various cellular pathways [103,104]. This study found that Twist1 connects to the proximal promoter of FOXA1 and draws the nucleosome remodeling deacetylase (NuRD) transcriptional repressor complex for the de-acetylation of histone 3 Lysine 9 (H3K9), which hinders the engagement of RNA polymerase II to strongly silence the transcriptional activity of the FOXA1 promoter [29]. Twist1 further mutes the FOXA1 promoter by obstructing the involvement of Activator Protein 1 (AP-1) [105,106,107]. A separate study suggested that Twist1 downregulates the expression of FOXA1 to increase the level of choline kinase in estrogen receptor-negative breast cancer cell lines, thus providing environmental advantages for cancer cells and promoting the metastasis of breast cancer [30]. In the context of basal-like breast cancer (BLBC), the abnormal presence of Twist1 induces EMT and properties akin to cancer stem cells, simultaneously suppressing ERα expression to enhance the resistance of breast cancer cells to chemotherapy drugs [31]. These all indicate that the suppression of FOXA1 expression by Twist1 plays a substantial role in the Twist1-driven migration, invasion, and metastasis of breast cancer cells. However, it has a lesser effect on the EMT morphology and the expression of certain EMT markers induced by Twist1 in these cells [29].

### 2.2. Signaling Pathway of FOXA2

FOXA2, which is highly similar to FOXA1 in the DNA-binding domain (DBD), is also considered to play an important role in the regulation of breast cancer. Previous studies have shown that FOXA2 can promote cell growth and sustain the presence of cancer stem cells in TNBC [94,108]. PGC-1β (Peroxisome proliferator-activated receptor-γ coactivator-1) is a key controller of mitochondrial creation and cellular metabolism and is often found to be overexpressed in the mammary gland [109]. Jia Cao et al. indicated that PGC-1β collaborates with FOXA2, leading to a reduction in the growth and movement of breast cancer cells [32]. Experimental results have revealed that a combination of PGC-1β downregulation and FOXA2 overexpression notably restrained the activity of breast cancer cells through the regulation of the PI3K-AKT-mTOR pathway [32,110]. Moreover, this pathway is also regulated by CD44. It is a kind of surface biomarker of cancerous stem cells (CSC) with the ability to enhance the stemness and metastasis of breast cancer through mesenchymal markers [111,112,113,114,115,116]. The study pointed out that CD44-mediated AKT phosphorylation promotes the nuclear translocation of FOXA2 [33]. Subsequently, CD44 can lead to FOXA2 accumulation in the cytosol and reduce E-Cadherin expression. This will indeed foster a mesenchymal characteristic, leading to an increase in the migration and invasion capabilities of cancer cells.

Generally, the regulatory impact of FOXA2 is often carried out in synergy with factors that have comparable effects [117] (Figure 2). For example, FOXP2, which is also a tumor suppressor, can interact with FOXA2 to inhibit the EMT of cancer cells [34,118]. This interaction can enable FOXP2, which is commonly considered a transcription inhibitor, to obtain transcriptional activity, thereby endogenously binding to the E-cadherin promoter and activating its transcription, as well as activating the expression of tumor suppressor PHF2 [34]. When the E-cadherin protein is activated, FOXA2 draws the transcriptional corepressor TLE3 to the promoter of ZEB2 to suppress its expression, which can effectively hinder the EMT of breast cancer cells [118,119,120,121].

### 2.3. Signaling Pathway of FOXC1

**Figure 3:**
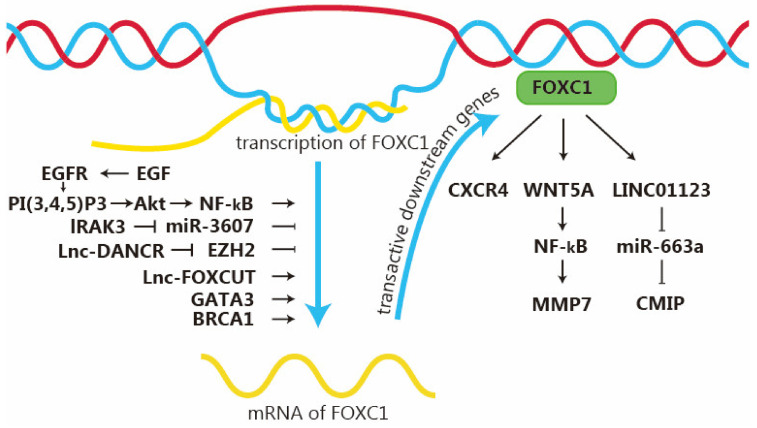
Regulation of FOXC1 and its oncogene effects. Oncogenic pathways mainly promote the transcription of FOXC1 to regulate its expression. Overexpressed FOXC1 acts as a transcription factor to transactive the expression of downstream oncogenes. Through the stimulation of the transcription of FOXC1, oncogenes can upregulate the downstream gene of FOXC1. Symbols: ↑ promote, ⊥ inhibit.

The FOXC1 gene is mapped to chromosome 6p25.5. Structurally, FOXC1 consists of an N-terminal transactivation domain, forkhead DNA-binding domain, transcription-inhibitory domain, and C-terminal transactivation domain [122,123]. In breast cancer, especially BLBC and triple-negative breast cancer (TNBC), FOXC1 plays an important role in promoting cell proliferation, migration, and EMT. Unlike the generally acknowledged tumor suppressor FOXA1, FOXC1 is considered to have dual roles in both oncogenesis and tumor suppression, with its specific behavior depending on its context within the pathway (Figure 3) [124,125]. Research has indicated that the breast cancer susceptibility gene 1 (BRCA1) and GATA3 can interact with the FOXC1 promoter to synergistically suppress the transcription of FOXC1 [35]. Therefore, the loss of GATA3 expression or irregular FOXC1 expression contributes to drug resistance and characteristics resembling EMT, which are related to aggressive BLBCs. Concurrently, another research study has shown that FOXC1 can compete with GATA3 in binding to the ER gene, which in turn contributes to the downregulation of ER [126]. However, unlike FOXA1, this indirect down-regulation of ER level, caused by competition between FOXC1 and GATA3, cannot inhibit the progression of breast cancer. This mechanism is used to explain the phenomenon of ER reduction in recurrent tumors after endocrine therapy.

Moreover, it was reported that under the catalysis of polycomb reactive complex 2 (PRC2), EZH2 can inhibit the transcription of its target gene through the trimethylation of Lys-27 on histone 3 (H3K27me3), thus controlling the expression of FOXC1 in breast cancer [37,127,128]. As early as 2006, Ingeborg M Bachmann et al. pointed out that EZH2 was associated with the high proliferation rate and invasion of breast cancer [129]. Subsequent studies by Alison Hirukawa have further proven that EZH2 sustains the suppression of the FOXC1 gene through H3K27me3, thereby deactivating an anti-invasive transcriptional program driven by FOXC1 in Luminal B breast cancer [130]. Furthermore, the stability of EZH2 might be under the control of O-GlcNAc transferase (OGT), an enzyme that can append GlcNAc units to specific proteins [131]. Although their research findings suggest that the regulation of FOXA1 and FOXC1 expression by OGT is not related to the stability of EZH2 protein, their migration/invasion assay results show that the expression level of OGT is directly proportional to the migration and invasion ability of cells. It is important to note that the influence of OGT on EZH2 is not consistent in mammalian cells because research by Myers and his team demonstrated that the reduction in OGT did not influence EZH2 and H3K27me3 in mouse embryonic stem cells [132]. Further research is needed on the mechanism of EZH2 regulation of FOXC1.

Although it is objectively recognized that FOXC1 plays a dual role in breast cancer, in actuality, its function in augmenting the invasiveness of breast cancer—particularly in triple-negative breast cancer (TNBC) and basal-like breast cancer (BLBC)—has been more extensively researched. In the upstream pathway of FOXC1, the EGF/NF-κB/FOXC1 signaling axis can play a significant role in BLBC [39]. The engagement of EGF can activate the EGFR receptor, which subsequently stimulates the pathways mediated by Ras or PI3K. Once Ras is activated, it leads to the phosphorylation and kinase activation sequence of RAF, MEK, and ERK. Meanwhile, PI3K phosphorylates PI(4,5)P2 and transforms it into PI(3,4,5)P3, which in turn activates Akt. Then, the activation of ERK and Akt results in the phosphorylation of NF-κB. Phosphorylated NF-κB moves into the nucleus and attaches to the promoter region of FOXC1, increasing the transcription and protein expression of FOXC1. The project team has already pointed out in previous research that FOXC1 can inversely enhance the activity and expression of NF-κB in BLBC cells, thereby augmenting cell proliferation, migration, and invasion [125].

Moreover, the role of long non-coding RNAs (lncRNAs) in regulating cell cycle, proliferation, and apoptosis in tumor development has been progressively uncovered in recent years. Concurrently, research into lncRNAs’ involvement in FOXC1 regulation has also commenced. A new study suggests that the disruption of the FOXC1/lnc-FOXCUT/lnc-DANCR axis might be a contributing factor to the aggressive characteristics of TNBC [40]. Lnc-FOXCUT is precisely positioned upstream of the FOXC1 promoter and FOXCUT has the function of targeting and stabilizing the FOXC1 transcript in EMT [133]. The FOXCUT–FOXC1 axis can trigger cancer invasion and metastasis either by activating the PI3K/AKT pathway or by enhancing the expression of matrix metalloproteinases (MMPs) [134,135,136]. Analogously, Lnc-DANCR and FOXC1 may potentially interact within the same EMT-related pathways, such as TGF-β and PI3K/AKT, suggesting an interconnected functionality [137]. However, another new theory suggests that the effects of these two may be achieved through EZH2. In their research, Zhang et al. demonstrated that lnc-DANCR promoted the binding of EZH2 to the promoter region of Suppressor of Cytokine Signaling 3 (SOCS3) [138]. This interaction led to the suppression of SOCS3 expression, thereby inducing malignant characteristics in breast cancer cells. The formation of the EZH2–DANCR complex may affect the binding of EZH2 transcription factors to their target gene promoter regions (e.g. FOXC1). Hence, as already discussed, lnc-FOXCUT and lnc-DANCR may contribute to TNBC malignant phenotypes [40].

For the downstream of the FOXC1 action pathway, the process of promoting breast cancer invasion is more complex, and more transcription factors are involved. According to the study by Purong Zhang et al., FOXC1-induced LINC01123 has a tumor-promoting effect in TNBC [41]. The upregulation of miR-663a exhibits anti-growth and pro-cell death impacts; however, the FOXC1-driven LINC01123 safeguards the oncogenic CMIP (c-Maf inducible protein) and encourages TNBC by restraining it [41,139,140]. H. Pan et al.’s research confirms that FOXC1 enhances the metastasis of TNBC by stimulating the transcription of CXC chemokine receptor-4 (CXCR4) [42]. CXCR4, a cell surface receptor with seven transmembrane-spanning domains that link to G protein, is significantly present in both initial and metastatic breast cancer cells and contributes to its metastasis [141,142,143]. The ChIP assay conducted by the researchers demonstrated the ability of FOXC1 to bind to the CXCR4 promoter, thereby enhancing its fold enrichment in breast cancer [42]. This is consistent with the previous experimental results of Hisaki Hayashi et al. [144]. CXCR4 is an upstream target of FoxC1, and its inhibition notably reduces the invasion and metastasis amplified by FOXC1 [42]. Similarly, research has demonstrated that the invasiveness of TNBC is regulated by FOXC1-driven non-canonical WNT5A–MMP7 signaling [43]. WNT5A is a ligand responsible for mediating non-canonical β-catenin-independent WNT signaling [145] in TNBC cells FOXC1; it binds to the WNT5A promoter region and significantly increased its expression. This resulted in the activation of NF-κB signaling, which subsequently led to the induction of matrix metalloproteinase-7 (MMP7) expression [43]. Overexpression of MMP7 can enhance the invasiveness of TNBC by disrupting the extracellular matrix [146].

FOXC2 is also found to be upregulated in breast cancer [147]. Overexpression of FOXO3 in BLBC is recognized as an inducer of EMT and metastasis [147]. Moreover, further research demonstrated that FOXF2 directly targets FOXC2 and represses the EMT of TNBC or BLBC [148].

### 2.4. Signaling Pathway of FOXF2

**Figure 4:**
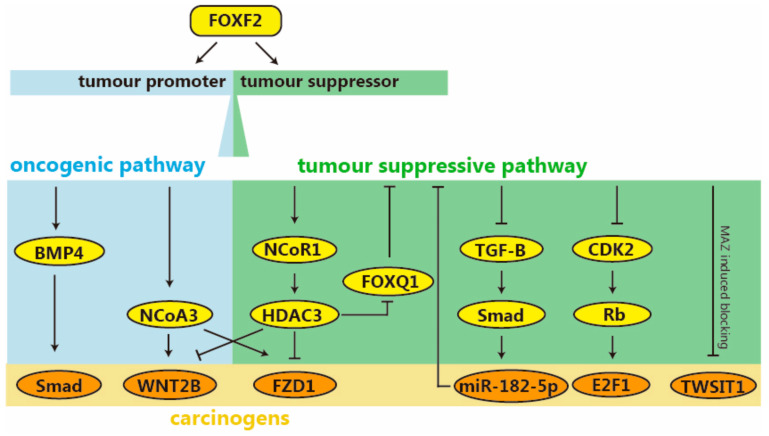
Oncogene effects of FOXF2. FOXF2 can act as both a tumor promoter and suppressor. The specific impact depends on the type of breast cancer and the particular situation. In general, FOXF2 exerts its dual function via oncogenic pathways and tumor-suppressive pathways. Symbols: ↑ promote, ⊥ inhibit.

Much like FOXC1, FOXF2 also exhibits a dual role in breast cancer, with its function significantly differing between the luminal and basal types of the disease (Figure 4). The FOXF2 gene is often inactive or “silenced” in luminal-type and HER2-positive breast cancers. However, it is overexpressed in basal-like breast cancers [46]. According to research by Xiao Zhang et al., FOXF2 enhances the stemness of luminal breast cancer cells through the Wnt/β-catenin pathway, but it reduces it in BLBC cells [45].

Nuclear receptor coactivator 3 (NCoA3), which is also amplified in breast cancer 1 (AIB1)/steroid receptor coactivator-3 (SRC-3), here, acts as a primary coactivator of FOXF2 in an ER-dependent fashion [149]. At the same time, nuclear receiver corepressor 1 (NCoR1) serves as the corepressor for FOXF2 and confers the functions of transrepressing on it. In luminal breast cancer cells, FOXF2 initiates the recruitment of NCoA3, forming a complex that attaches to the WNT2B and FZD1 promoters. This combination finally enhanced the transcriptional activity of WNT2B and FZD1 promoters [150]. On the contrary, FOXF2 does not recruit NCoA3 but instead recruits NCoR1and histone deacetylase 3 (HDAC3) to bind to the promoters of WNT2B and FZD1, which ultimately inhibits the transcriptional activity of the promoter in BLBC. Therefore, in luminal breast cancer and basal-like breast cancer cells, FOXF2 differentially modulates the transcription of WNT2B and FZD1 by enlisting NCoA3 and NCoR1, respectively.

In luminal and HER2-positive breast cancer, FOXF2 is believed to play a major role in regulating the cell cycle. The study by Pang Kuo Lo and colleagues suggests that the abnormal expression of FOXF2 can hinder the CDK2-Rb-E2F cascade, causing cells to be stuck in the G1 phase [46]. Phosphorylation of CDK2 at Thr160 facilitates the transition from G1 to S phase, whereas FOXF2 acts as a suppressor of CDK2 activation [151]. This suppression, in turn, hinders the phosphorylation of RB protein mediated by CDK2. At the same time, phosphorylation of RB protein can relieve the inhibition of E2F1, allowing E2F1 to promote cell cycle progression [152]. Therefore, the effect of FOXF2 ultimately leads to G1 phase arrest and induces apoptosis of cancer cells. Additionally, the study also explores the frequent silencing of FOXF2 in luminal and HER2-positive breast cancer, attributing it to the hypermethylation of the FOXF2 CpG-island-containing promoter.

In BLBC, the carcinogenic mechanism of FOXF2 is often related to its expression level. The overexpression of FOXF2 inhibits basal-like breast cell proliferation, whereas a deficiency of FOXF2 increases the aggressiveness of BLBC cells by bestowing upon them properties similar to mesenchymal stem cells [48,153]. The actions of FOXF2 in BLBC cells are akin to those of FOXA1 in luminal breast cancer. According to a study published by Wang Shuo et al., in 2019, FOXF2 can promote the epithelium-to-osteomimicry transition (EOT) of breast cancer cells by activating BMP4/Smad1 signaling pathway, thus reprogramming them into bone metastasis seeds, and ultimately promoting the bone metastasis tendency of cancer cells [47]. The Bone Morphogenetic Protein (BMP) signaling pathway is critically implicated in the bone metastasis of breast cancer [154]. This study suggests that the FOXF2 protein binds to the BMP4 and Smad1 promoter regions to positively regulate its expression.

In fact, FOXF2 has a similar effect on visceral metastasis in addition to playing a role in bone metastasis of breast cancer. According to Jun Tao Lu et al., by significantly enhancing TGF-β and miR-182-5p, FOXF2 deficiency expedites the spread of BLBC to internal organs [48]. Research has shown that BLBC cells lacking FOXF2 are inclined to spread to visceral organs. (This has also been proven in the research of Wang et al., which was mentioned earlier.) This is facilitated by the amplification of autocrine TGF-β signaling, which also imparts aggressiveness to proximate cells through the enhancement of paracrine TGF-β signaling [155,156]. In response, TGF-β suppresses FOXF2 expression by elevating miR-182-5p (a post-transcriptional controller of FOXF2 and a promoter of metastasis) [48]. The expression level of miR-182-5p is inhibited by FOXF2, but mothers against decapentaplegic homolog 3 (Smad3) can activate it by binding to its promoter. Overall, FOXF2 acts as an inhibitor for both TGF-β and miR-182-5p and is deactivated by the TGF-β/SMAD/miR-182-5p pathway; FOXF2 deficiency expedites the visceral metastasis of BLBC through the uncontrolled elevation of both autocrine and paracrine TGF-β signaling, as well as the expression of miR-182-5p.

In addition, FOXF2 can also act on the invasiveness of BLBC through other pathways. Kang et al. pointed out that the aggressiveness of BLBC can be regulated by reciprocal transrepression between FOXF2 and FOXQ1 [150]. Researchers have demonstrated, through a series of experiments, such as dual luciferase reporter assays, that FOXF2 draws NC oR1 and HDAC3 to the FOXQ1 promoter, thereby suppressing its transcription. Although FOXQ1 cannot exert a reverse effect on FOXF2 through this pathway, it can still inhibit the expression of FOXF2 and form a mutual negative feedback loop. The increase in basal-like breast cancer aggressiveness is linked to the disruption of this negative feedback mechanism. Furthermore, it is worth mentioning that the total expression levels of FOXF2 and FOXQ1 in the cells and tissues did not seem to have a negative correlation.

### 2.5. Signaling Pathway of FOXM1

**Figure 5:**
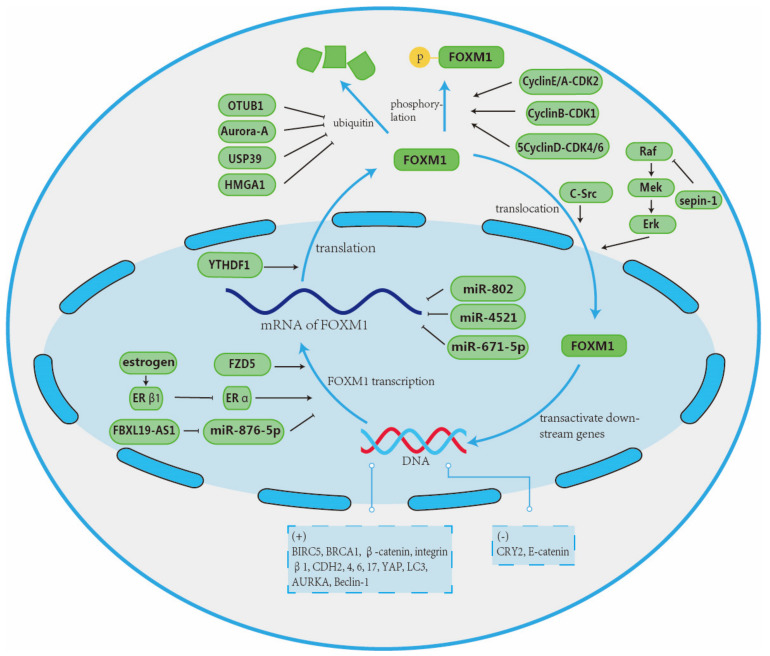
Regulation of FOXM1 and its oncogene effects. Dysregulated factors target FOXM1 and eventually induce the deterioration of cancer. Overexpression of FOXM1 tends to transactive many oncogenes, which promote the proliferation, EMT, and metastasis of breast cancer. Mechanisms regulating FOXM1, including translation, translocation, phosphorylation, and ubiquitin. Symbols: ↑ promote, ⊥ inhibit.

During the progression of breast cancer, various dysregulated factors target FOXM1 and eventually induce the deterioration of the cancer. The overexpression of FOXM1 tends to transactive many oncogenes, which promote the proliferation, EMT, and metastasis of breast cancer (Figure 5).

The 6-methyladenosine (m^6^A) is the most common internal modification targeting messenger RNA (mRNA), which alters the structure of RNA via methylation and affects its interaction with the m^6^A binding protein (reader) [157]. Studies have shown that YTH N6-Methyladenosine RNA Binding Protein 1 (YTHDF1) serves as a reader and targets the mRNA of FOXM1 [158,159]. It directly binds to the m^6^A-modified mRNA of FOXM1 and enhances the translation process of FOXM1 [49]. Therefore, the overexpression of YTHDF1 in breast cancer cells largely augments the expression of FOXM1 and promotes breast cancer cell proliferation, invasion, and EMT.

Long-non-coding RNAs (lncRNAs) are a kind of non-coding RNA that transcript more than 200 nucleotides that function as gene regulators [160,161]. It is demonstrated by large amounts of research that lncRNA is dysregulated in breast cancer [162]. Dong G et al. discovered that F-box and leucine-rich repeat protein 19 antisense RNA 1 (FBXL19-AS1) is a lncRNA and serves as an oncogene in breast cancer via miR-876-5p/FOXM1 axis: miR-876-5p was reported as a tumor suppressor which represses the expression of FOXM1 [163,164], and FBXL19-AS1 overexpression circumvents the function of miR-876-5p via directly absorbing it in the cytoplasm, thereby upregulating the expression of FOXM1 to induce breast cancer [50].

Ubiquitination is a reversible process that is characterized by the binding of ubiquitin. Target protein marked by ubiquitin acquires regulatory functions such as degradation, change in activity, and change in location. For instance, it was indicated that ubiquitination E3-ligase RNF 168 is involved in DNA damage response. It can restrict breast cancer cell proliferation and enhance the treatment effectiveness of epirubicin by targeting FOXM1. As FOXM1 confers breast cancer cells’ phenotype of epirubicin resistance by repairing DNA damage, RNF 168 degrades FOXM1 through ubiquitination with the help of RNF8 [51].

In contrast, the deubiquitinating process can commonly stabilize the structure of the target protein. It has been confirmed that deubiquitinating enzyme OTUB1 can protect target proteins from ubiquitination in response to DNA damage [165]. Karunarathna U et al. demonstrated that OTUB1 can bind to and stabilize FOXM1 in epirubicin-treated breast cancer cells, which enhances its role in DNA damage response and epirubicin resistance of breast cancer cells [52]. Similarly, in TNBC, aurora kinase A (Aurora-A) can also prevent FOXM1 from ubiquitination, therefore, stabilizing the structure and oncogenic effect of FOXM1. The overexpression of Aurora-A promotes TNBC cell proliferation and resistance to paclitaxel [53]. Additionally, the deubiquitinating enzyme USP39 can also directly bind to FOXM1 and further deubiquitinate and stabilize FOXM1. Furthermore, FOXM1 can upregulate USP39, therefore forming a positive feedback loop that promotes breast cancer cell proliferation [54].

MicroRNAs (miRNAs) are small non-coding RNA molecules that are composed of 20–25 nucleotides [166]. Research has discovered that miRNAs play a dual role in cancer development, which depends on the specific miRNA; some have an oncogenic effect, while others have roles as tumor suppressors [167,168]. As the upstream gene of FOXM1, miRNA-4521 directly targets FOXM1 in breast cancer cells and inhibits its expression, inducing cell cycle arrest, DNA damage, and cell death [55]. Similarly, Tan X et al. demonstrated that miR-671-5p also functions as a tumor suppressor via directly binding to FOXM1 and downregulating its expression. In detail, overexpression of miR-671-5p can reverse EMT to MET in breast cancer cells and restrict the cell cycle in the S-phase. Moreover, upregulated miR-671-5p can enhance the effect of drugs such as cisplatin, 5-fluorouracil, and epirubicin by dampening the drug-resistance effect of FOXM1 [56]. Additionally, Yuan F et al. also found that miR-802 can inhibit breast cancer cell proliferation by directly targeting FOXM1 [57].

Millour J et al. discovered that in ER-positive breast cancer cells, ER transactivates FOXM1 via an estrogen-response element (ERE) in the proximal promoter region [58]. Additionally, Horimoto Y et al. found that ERβ1 can also bind to FOXM1 promoter in ERE which competes with ER for binding site. So, upregulated ERβ1 diminishes the promoting effect of ER and the oncogenic effect of FOXM1 [59].

FOXM1 is also regulated by another post-transcriptional modification known as phosphorylation. Studies have confirmed that phosphorylation is one of the most common modifications occurring in breast cancer that determines the activation state, cellular localization, and stability of FOXM1 [62,64]. Nandi I et al. revealed that in luminal B breast cancer, c-Src phosphorylated FOXM1 on 2 tyrosine residues, and promoted its nuclear localization and, subsequently, target gene expression. Except for targeting G2/M phase genes to promote breast cancer cell proliferation, FOXM1 transactive c-Src forms a positive feedback loop that leads to uncontrolled proliferation and breast cancer [169,170,171]. It is reported that the Raf/Mek/Erk cascade phosphorylated FOXM1 and leads to FOXM1 nuclear translocation, which activates FOXM1 [60]. Additionally, Zhang N et al. reported that Sepin-1 inhibits breast cancer cell proliferation via the downregulation of FOXM1 in the manner of the Raf/Mek/Erk pathway [61].

As a main regulator of G1/S and G2/M cell cycle transitions, FOXM1 is regulated by various kinases associated with the progression of the cell cycle. Research has revealed that during the G1/S phase, CyclinD-CDK4/6 phosphorylates FOXM1 in the C-terminal region. Phosphorylation can stabilize the structure of FOXM1 and promote its effect of proliferation [62]. Similarly, Major ML et al. confirmed that during the G1/S phase, CyclinE/A-CDK2 and CyclinB-CDK1 bind to the N terminus of FOXM1b and elevate its transcriptional activation via the recruitment of co-activator [63,64].

Zanin R et al. revealed that in TNBC, High Mobility Group A1 (HMGA1) regulates FOXM1 at the post-transcriptional level by preventing it from proteasomal degradation in the nucleus. FOXM1 also serves as a molecular partner of HMGA1 in breast cancer progression: HMGA1 binds to FOXM1 and increases its activity as a promotor of Vascular Endothelial Growth Factor (VEGFA). As the downstream gene, VEGFA is the main inducer of angiogenesis, and the cooperative effect of HMGA1 and FOXM1 plays an important role in TNBC progression [65].

FOXM1 overexpression usually occurs in breast cancer and is always correlated with poor prognosis. As a master regulator of G1/S and G2/M cell cycle transitions, FOXM1 promotes cancer progression mainly by accelerating cell cycle and proliferation [172,173,174]. FOXM1 can also exert its oncogenic effects by promoting EMT, angiogenesis, invasion, metastasis, drug resistance, and so on.

As reported, FOXM1 contributes to EMT by augmenting the transcription of mesenchymal-specific transcription factors, such as slug [66]. Slug is known as a zinc-finger protein that represses the expression of E-cadherin cell–cell adhesion protein [175]. So, the FOXM1-induced overexpression of slug confers invasive phenotypes to breast cancer cells and stimulates EMT. Additionally, Li Z et al. discovered that FOXM1 can promote EMT through FOXM1/KIF23/Wnt/β-catenin pathway: kinesin family member 23 (KIF23) is the direct target of FOXM1, whose overexpression increases KIF23 levels in TNBC cells. The overexpression of KIF23 subsequently activates the Wnt/β-catenin pathway, which contributes to TNBC cell EMT. Scientists have also found that this pathway was accelerated by WDR5, which accelerates the histone modification function of H3K4me3 to FOXM1 and promotes FOXM1 expression [67].

In TNBC, FOXM1 was found to upregulate the expression of the ITGB1 gene that encodes integrin β1 by physically binding it to the promotor [176]. Integrin β1 upregulation always leads to cellular proliferation, migration, invasion, and resistance to apoptosis, which plays an important role in TNBC progression [68]. Furthermore, eukaryotic elongation factor 2 kinase (eEF-2K) expression is promoted by integrin β1 or the direct binding of FOXM1 [176]. The upregulation of eEF-2K is always associated with TNBC cell proliferation and invasion, which leads to tumor growth and progression [177]. In ER+ breast cancer cells, FOXM1 engages in core circadian gene cryptochrome 2 (CRY2) promoter hypermethylation by forming the FOXM1/DNMT3b (DNA methyltransferase 3b) complex; the FOXM1/DNMT3b complex binds to the CRY2 promoter directly to downregulate the expression of CRY2 [69]. Then, the dysregulation of the CRY2 leads to an increase in breast cancer risk and aggressiveness [178,179,180].

It is reported that breast cancer stem cells (BCSCs) play a pivotal role in breast cancer initiation, recurrence, and drug resistance [181]. Sun Y et al. reported that as a member of the Frizzled family, Frizzled5 (FZD5), can exacerbate TNBC by facilitating breast cell proliferation, DNA damage repair, and stemness. Researchers found that TNBC cells with FOXM1 overexpression and low levels of FZD5 show a FZD5-associated phenotype. Further experiments proved that FOXM1 is a downstream factor of FZD5: FZD5 can activate the Wnt/β-catenin pathway to increase the expression of FOXM1, which in turn elevates the expression of BRCA1 and BIRC5 by binding to their promoters [70]. Moreover, the maintenance of stemness of BCSCs is reported to be correlated with the co-work of FOXM1 with cAMP-response element-binding (CREB) binding protein (CBP) [71] and cadherins (CDHs) [72]. In TNBC, FOXM1 binds with CBP/β-catenin to constitute a transcriptionally active complex, leading to increased CSC numbers and stemness [71]. FOXM1 also regulates CDH to keep TNBC cells stemness: as a stem cell-related transcription factor (SC-TF), FOXM1 upregulates CDH2,4,6, and 17, which is correlated with the maintenance of stemness in TNBC [72]. Hong-Liang Sun et al. discovered that the YAP1 pathway increases breast cancer cell stemness by promoting the expression of OCT-4 and NANOG expression in TNBC [182]. Additionally, the overexpression of FOXM1 prevents the phosphorylation of YAP1 and subsequently upregulates YAP1 expression, therefore promoting cancer cell proliferation, migration, and stemness [73], N Yang et al. reported that there exists a positive feedback between nuclear aurora kinase A (AUPKA) and FOXM1 in BCSCs, which is essential for the maintenance and self-renewal of BCSCs [74], Specifically, AURKA, as a transactivating co-factor, transcriptionally activates FOXM1 expression independent of its kinase activity. Meanwhile, FOXM1 directly binds to the AURKA promoter and upregulates its expression [74]. Consequently, AURKA and FOXM1 collaboratively contribute to the aggravation of breast cancer. Moreover, AURKA itself plays a significant role as a kinase that promotes breast cancer proliferation and metastasis of breast cancer cells [183,184].

Autophagy, as a degradation mechanism mediated by lysosomes, plays a dual role in both the suppression and progression of cancer [185]. In the late stage of tumor progression, autophagy serves as a survival pathway that captures, degrades, and recycles intracellular proteins and organelles to facilitate cancer cells’ survival from microenvironment stress and promote tumorigenesis [186]. When TNBC cells are subjected to stress, FOXM1 was found to promote autophagy by directly binding to the promotor of autophagy-related genes such as microtubule-associated light chain 3 (LC-3) and Beclin-1 during autophagy induction [75]. Therefore, FOXM1 can mitigate stress in TNBC cells by enhancing the autophagy process.

FOXM1 also exerts its oncogenic effects via regulating miRNA expression. Hamurcu Z et al. discovered that FOXM1 can upregulate miR-186-5p and downregulate miR-200b-5p, leading to adverse outcomes in TNBC cells: the miR-186-5p is recognized as an oncogenic miRNA that promotes cancer cells progression by regulating Beclin-1. On the other hand, miR-200b-5p has a tumor repression effect as it is capable of inducing cell cycle arrest. Researchers have also discovered many other miRNAs that are regulated by FOXM1 and play crucial roles in important pathways in breast cancer, such as miR-21, miR-30a, and miR-95 [76,187,188].

### 2.6. Signaling Pathway of FOXO3

**Figure 6:**
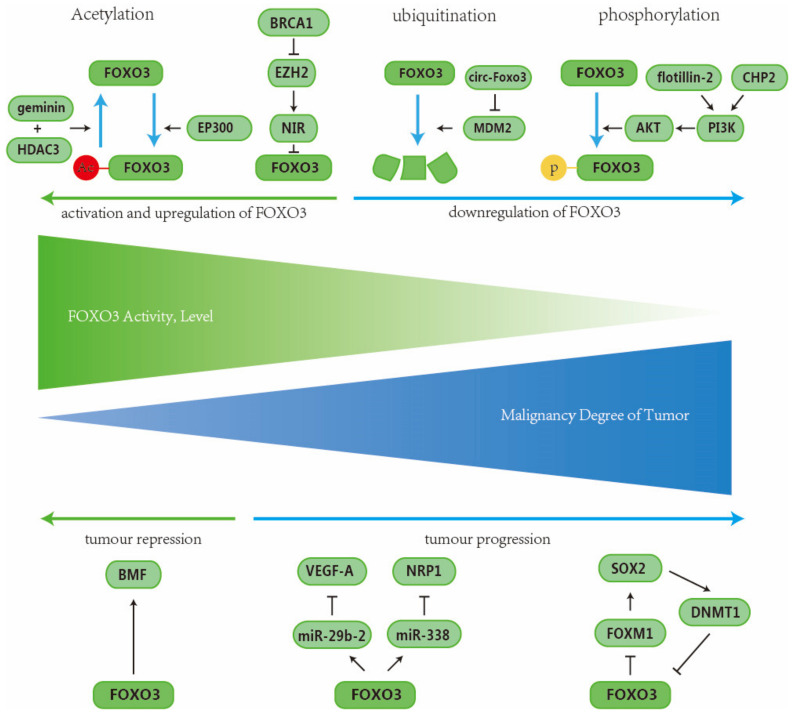
Regulation and tumor suppressive effects of FOXO3. The activity and level of FOXO3 are negatively related to the malignancy degree of breast cancer. The pathways above the figure regulate FOXO3 activity and level. The specific mechanism includes acetylation, ubiquitination, and phosphorylation. The pathways below the figure illustrate how FOXO3 represses breast cancer. FOXO3a represses breast cancer by promoting the expression of tumor suppressors and repress the expression of oncogenes. Symbols: ↑ promote, ⊥ inhibit.

FOXO3 is commonly known as a tumor suppressor, and its expression level and activity are negatively related to the malignancy of breast cancer. During the prognosis of breast cancer, FOXO3 tends to be repressed and inactivated. Detailed mechanisms are introduced below (Figure 6).

Gong C et al. discovered that there is a significant correlation between the decreased expression of FOXO3 and the deficiency of BRCA1. This association is attributed to the mechanism involving FOXO3 promotor methylation: as zeste homologue2 (EZH2) can facilitate the recruitment of DNA methyltransferase to the promotor of the FOXO3 gene, the FOXO3 gene is subsequently methylated, and the expression is repressed. Therefore, BRCA1 can release FOXO3 gene methylation by downregulating EZH2. This, in turn, leads to the reversal of the downregulation of FOXO3 in breast cancer [36]. Additionally, Chen B et al. made an observation that EZH2 can also recruit the Novel INHAT Repressor (NIR) to the promoter vicinity of FOXO3 via protein–protein interaction. The binding of NIR in the promoter vicinity of FOXO3 can also restrain the function of FOXO3 through histone H3 lysine 27 (H3K27) acetylation and trimethylation [38]. To summarize, the silencing of both EZH2 and NIR can boost the expression of FOXO3 and suppress breast cancer cell proliferation.

Circular RNAs (circRNAs) are a type of non-coding RNA characterized by their closed loop-like structure that is predominantly found in the cytoplasm [189,190]. They act as miRNA sponges, thus inhibiting the functions of miRNAs. They can also function as scaffolds, binding to proteins and promoting protein–protein interaction. [189,191,192]. CircRNA circ-Foxo3s have been identified as a tumor suppressor that participated in breast cancer cell apoptosis and inversely correlated with breast cancer progress [193]. For instance, Du WW et al. discovered that circ-Foxo3 can induce cancer cell apoptosis by preventing FOXO3 from ubiquitination: as a main target of MDM2, FOXO3 can be ubiquitinated and degraded by binding to MDM2 [77]. However, when circ-Foxo3 binds to MDM2, it has a low affinity with the FOXO3 protein, thus preventing the interaction between MDM2 and FOXO3. As a result, the ubiquitination and degradation of FOXO3 are inhibited [194].

Acetylation is a crucial post-transcriptional modification that can alter the activity and affinity of FOXO3 for DNA, resulting in either a decrease or increase in its function [195,196]. There is evidence that suggests that the acetylation of FOXO can trigger cell apoptosis and increase the nuclear concentration of FOXO protein. [195,196]. For instance, Mahmud Z et al. demonstrated that the interaction between EP300 and FOXO3 leads to FOXO3 gene acetylation, ultimately facilitating lapatinib cytotoxicity in lapatinib-sensitive HER2-positive breast cancer. Lapatinib treatment also promotes EP300–FOXO interaction and FOXO3 recruitment, in turn [78]. On the other hand, deacetylation of FOXO3 may block its activity. For example, Zhang L et al. discovered that geminin could facilitate breast cancer cell metastasis via mediating FOXO3 deacetylation: geminin selectively forms a complex with the histone deacetylase HDAC3 and FOXO3, leading to the deacetylation of FOXO3. Geminin-mediated FOXO3 deacetylation attenuates FOXO3 transcriptional activity. This process results in the attenuation of FOXO3’s transcriptional activity and subsequently leads to the downregulation of its downstream target, Dicer [197]. As Dicer is an RNase that suppresses metastasis [79], geminin facilitates breast cancer cell metastasis via the geminin/HDAC3/FOXO3/Dicer pathway.

Studies have discovered that the dysregulation of PI3K/AKT pathway signaling is closely related to poor prognosis in breast cancer patients [198,199]. To explain the potential mechanism, Greer EL et al. conducted an experiment and revealed that the PI3K/AKT pathway can exert its oncogenic effort via downregulating FOXO3a level: AKT promotes the phosphorylation of FOXO3a and leads to the translocation to the cytosol and degradation [200]. In the case of BCSCs, via targeting FOXO3a, the PI3K/AKT pathway can keep BCSC features such as mammosphere formation, inhibition of differentiation, and recurrence of breast cancer [201]. Furthermore, Xie G et al. revealed that flotillin-2 can promote the proliferation of breast cancer cells via activating the PI3K/AKT pathway and subsequently phosphorylate FOXO3a [80]. Similarly, X. Zhao found that the overexpression of CHP2 also suppresses the transactivation of FOXO3 by activating AKT signaling [202]. Zhang H et al. indicated that miR-940 directly binds to FOXO3 and downregulates its expression, therefore promoting the malignant progression of breast cancer [81].

FOXO3 has been identified as a suppressor of breast cancer, as it can inhibit tumor progression and suppress BCSC properties. This remarkable effect is attributed to its capacity to bind to and regulate the promoters of specific target genes as a transcriptional factor [203]. Studies have revealed that the high expression of FOXO3a serves as an optimistic prognostic marker. It has been found to exhibit a negative correlation with the oncogenic pathways such as PI3K/AKT/mTOR while showing a positive correlation with apoptosis signaling P53 [204]. The downregulation of the FOXO3 gene and the subsequent decrease in FOXO3 protein levels are commonly observed in breast cancer cells [205]. Research indicates that the regulation of breast cancer progression and properties of BCSCs is influenced by FOXO3a. Specifically, FOXO3a competes with FOXM1 for binding to the promoter site of SOX2. This competition plays a crucial role in modulating the behavior of breast cancer and BCSCs. As mentioned earlier, FOXM1 is responsible for the maintenance of BCSCs stemness and promotion of breast cancer progression [74]. This is achieved through binding to and upregulating the expression of SOX2 [206]. Conversely, FOXO3a antagonizes the FOXM1 oncogenic function in the manner of the SOX2 mechanism. Unfortunately, SOX2 can initiate a feedback loop wherein it represses FOXO3a expression via direct transactivating DNMT1 expression in breast cancer cells. DNMT1 subsequently methylates the FOXO3a gene, resulting in the downregulation of FOXO3a expression. Ultimately, this dysregulation of FOXO3a contributes to breast cancer progression and poor prognosis [82].

Anoikis is a form of programmed cell death that occurs when cells become detached [207]. Hornsveld M et al. have discovered that anoikis can be stimulated by increasing FOXO3 in breast cancer cells that lack the E-cadherin protein. This activation occurs through the direct transactivation of the pro-apoptotic protein BMF by FOXO3. Moreover, in breast cancer cells, E-cadherin inactivation may lead to FOXO3 inhibition in a PI3K/AKT-dependent manner. In conclusion, increased FOXO3 and decreased E-cadherin in breast cancer cells can induce anoikis through direct activation of BMF [83]. Additionally, research has provided evidence that the signaling pathway involving vascular endothelial growth factor A (A) and neuropilin 1 (NRP1) plays a crucial role in promoting metastasis and invasion of breast cancer cells, which is often associated with a poor prognosis [208,209,210]. A study conducted by Song Y et al. further revealed that FOXO3 governs the expression of two microRNAs, miR-29b-2 and miR-338, which directly target VEGF-A and NRP1, respectively, leading to the suppression of their expression. Consequently, the tumor-suppressive role of FOXO3 is substantiated by its ability to increase the levels of these miRNAs, ultimately resulting in the repression of the oncogenic factors VEGF-A and NRP1 [84].

### 2.7. Other FOX TFs

There are also many FOX family members who have not been introduced in detail. Those FOX TFs also play a significant role in the progression of breast cancer. Their function and rude mechanisms are introduced in the following content.

FOXK2 commonly functions as a tumor suppressor. While ERα is a crucial regulator of breast cancer progression, FOXK2 can repress its function via enhancing ERα ubiquitin-mediated degradation [211]. Associated with multiple transcription corepressor complexes, FOXF2 can repress the tumorigenesis and hypoxic response of breast cancer, and during the progression of breast cancer, FOXK2 expression is progressively lost [212].

FOXP2 plays a dual role in breast cancer development. Initially, FOXP2 functions as a tumor suppressor, targeting various tumor inhibitors such as BAX, PTEN, and p16 [147]. The overexpression of FOXP3 is related to the upregulation of apoptosis-related factors such as programmed cell death 4, which can lead to breast cancer cell death [147].

The expression of FOXQ1 varies across different types of breast cancer. Studies indicate that its expression is reduced in luminal and HER2-positive breast cancer [213]. In contrast to other cancers like colorectal cancer, where low FOXQ1 expression often signifies a favorable prognosis, the opposite is true for breast cancer. This discrepancy could be attributed to the role FOXQ1 plays in the EMT process in breast cancer. Research by Yuan Qiao et al. suggests that FOXQ1 triggers the EMT process in breast epithelial cells, and its overexpression is linked to chemotherapy resistance in breast cancer [214]. Further studies have identified Twist1, Zeb2, and PDGFR α and β as downstream targets of Foxq1. Inhibiting PDGFR α and β has been shown to partly counteract the tumor-promoting effects of FOXQ1, offering potential new avenues for reducing drug resistance and developing treatment strategies for breast cancer [215].

## 3. Treatment

As the most common cancer among women, the treatment of breast cancer has been extensively studied in the past decades, and breast cancer also has its main treatment methods according to different classifications [216]. For hormone receptor-positive breast cancer, such as ER+ and PR+, the mainstream treatment method is endocrine therapy [217]. Take tamoxifen as an example, its principle is to competitively inhibit the combination of estrogen and ER to offset the promotion of estrogen on tumors [218]. For HER2-positive breast cancer, chemotherapy and targeted drugs are the current standard treatment scheme. Representative drugs include anti-HER2 monoclonal antibodies such as trastuzumab and pertuzumab, as well as lapatinib, which blocks the HER2 and EGFR pathways [219]. For TNBC, the current treatment methods are chemotherapy (such as platinum and paclitaxel drugs) and targeted drugs, such as pembrolizumab, which can be used in combination with chemotherapy drugs [220].

However, a large number of studies have shown that, although the current standard treatment for breast cancer has achieved remarkable results, the problem of drug resistance related to it has gradually become prominent [221]. We found that the FOX TF is closely related to the mechanism leading to drug resistance in breast cancer, so here we list some mechanisms that have been found at present and hope to find new ways for the treatment of breast cancer (Table 2).

### 3.1. Relative Drugs

#### 3.1.1. Telaprevir

A recent study suggests that the adjustment of ERα internal quantities can serve as a lure for recognizing new compounds that either directly or indirectly influence ERα amounts and operations, thereby inhibiting the advancement of breast cancer [228]. Telaprevir (Tel) is an oral NS3/4A protease inhibitor, which is routinely used to treat hepatitis C virus infection and is not considered to be associated with breast cancer [229]. However, a new study proves that Tel prompts cellular demise by diminishing the expression of FOXA1 in estrogen receptor α (ERα)-positive breast cancer cells through the IGF1-R/AKT/FOXA1 pathway [222]. Stefania Bartoloni et al.’s cell experiments showed that Tel-induced caspase-dependent apoptosis only occurs in cell lines expressing FOXA1 mRNA, especially MCF-7 cell lines, and has little effect on untransformed cell lines. Additionally, Tel can inhibit the activity of FOXA1 on its specific enhancer in the ESR1 promoter, and FOXA1 is required for Tel’s ability to prevent cell proliferation [94,230]. Much like ET drugs currently employed in breast cancer treatment, Tel exhibits suppressive effects on E2:ERα signaling (ERα is the main driver of E2 mitogenic stimuli) [231]. These results indicate that Tel may serve as a supplementary medication for managing primary and metastatic breast cancer treatment. It is worth noting that although Tel is a drug approved by the FDA for the treatment of hepatitis C, it is still in the preclinical stage in the treatment of breast cancer.

#### 3.1.2. Pyrotinib

The study by Chaokun Wang et al. suggests that in HER2-positive breast cancer, pyrotinib (PYR) combined with adriamycin (ADM) has significant anti-tumor effects [232]. The cell lines used in this experiment are breast cancer cell lines SK-BR-3 and AU565. PYR can irreversibly inhibit multiple ErbB receptors, and it is a comprehensive HER kinase suppressor that hinders signaling through the PI3K/AKT and RAS/RAF/MEK/MAPK pathways [223,233]. In this study, their research indicated a notable decrease in Akt, p-65 phosphorylation, and FOXC1 protein levels in breast cancer cells following PYR treatment. However, adriamycin did not exhibit a similar impact in experimental cells [232]. These results suggest that PYRimpedes cell growth, movement, and invasion by deactivating the Akt/p-65/FOXC1 signaling pathway in HER2-positive breast cancer cells, consistent with previous research findings [234]. Generally, this combination therapy may be more effective than PYR or adriamycin alone. Currently, PYR is in phase III clinical trials and has not yet been put into formal use [235].

#### 3.1.3. N-3(C_23_H_17_O_9_N_2_Cl)

Recently, researchers have created new bifendate derivatives, which have shown the potential to inhibit the migration and invasion of TNBC cells in a laboratory setting [236,237]. A recent study suggests that N-3, a novel synthetic bifendate derivative, reduces the metastasis of TNBC by diminishing the stability of the FOXC1 protein, which is regulated by p38 [224]. The cell lines used in this experiment are MDA-MB-231 and 4T1 cells. Additionally, this research indicates that N-3 enhances proteasome degradation through ubiquitin, resulting in a decrease in FOXC1 protein stability. The regulation of FOXC1 could potentially serve as an innovative and targeted treatment approach for metastasis in TNBC. At present, the drug has only a theoretical basis and has not yet entered the clinical trial stage.

#### 3.1.4. NB-55

As a crucial breast cancer oncogene, FOXM1 has been profoundly studied, and many compounds that inhibit FOXM1 have been found. Y. Ziegler et al. discovered a new compound class that directly binds FOXM1 via high-throughput screen and FOXM1 target engagement verification. They are 1,1-diarylethylene mono-and diamine compounds, and the first lead compound is NB-55. NB-55 functions as the FOXM1 suppressor, which directly binds to FOXM1 and makes it more liable to be degraded by protease. Further investigation validated that those compounds significantly suppress breast cancer cell proliferation and promote apoptosis, guaranteeing patients a better prognosis in TNBC [238,239]. This experiment used various cell lines such as MCF-7, T47D, MDA-MB-231, etc. In addition, the current research on NB-55 is still in the preclinical stage and has not yet undergone clinical trials.

#### 3.1.5. CRBN-Recruiting Molecule

G. Luo and colleagues designed a novel molecule called CRBN-recruiting molecule, which is acquired by silico modeling. This molecule is designed to bind to the CRBN (Cullin-4-Ring Box), the E3 ubiquitin ligase component, and has a high facility for FOXM1 protein. So, this molecule is capable of recruiting FOXM1 protein to CRBN and promoting FOXM1 protein ubiquitination and degradation in the MDA-MB-231 cell model. This FOXM1-targeting chimera has proved its ability to degrade the FOXM1 level and has the potential to repress the TNBC progression [226]. At present, the drug is still in the preclinical stage and has not yet undergone clinical trials.

#### 3.1.6. Disulfiram (DSF)

DSF has been put into clinical practice as an alcoholism drug. However, its effect on anti-breast cancer is receiving more and more attention. The underlying mechanisms have been deeply analyzed, and their effect on FOXO has been revealed. As mentioned above, AKT can phosphorylate FOXO3a and promote its translocation and eventually degradation. As DSF reduces the level of p-AKT, it indirectly prevents the FOXO3a from phosphorylation in breast cancer. The cell lines used in this experiment are MCF-7, MDA-MB-231, and HBT-3 cells. And this study also found that DSF can make breast cancer cells sensitive to chemotherapy drugs (such as paclitaxel and cisplatin) to a certain extent. In conclusion, DSF has anti-proliferation activity, which is achieved via the regulation of the AKT–FOXO axis [227]. Although DSF has been approved by the FDA to treat alcoholism, it has not been fully studied as a drug for breast cancer.

### 3.2. Drug Resistance

Although many FOX TFs have been proven to be related to the treatment of breast cancer or may become potential targets, some studies have pointed out that FOX TFs are related to drug resistance of some drugs, especially non-targeted drugs used early. Tamoxifen is a selective estrogen receptor modulator, which is widely used in the treatment of ER-positive breast cancer [240]. However, recent studies have pointed out that invasive lobular breast cancer (ILC) has higher tamoxifen resistance than invasive ductal carcinoma (IDC) [241]. Agostina Nardone et al.’s experiment suggests that this resistance is due to the abnormal impact of ILC’s unique chromatin state on the FOXA1–ER axis. Their transportase accessible chrome sequencing (ATAC seq) record showed that the top enriched motifs in the ILC gained sites were FOXA1 motifs, which makes ILC closely related to FOXA1 recruitment. Since FOXA1 promotes ER binding, this may weaken the effect of tamoxifen, leading to drug resistance.

In addition to resistance to endocrine therapy, the FOX family plays a role in chemotherapy drug resistance through different mechanisms. FOXC1 passes MicroRNA-495/TGF in triple negative metaplastic breast cancer (MBC)-β; the FOXC1 axis is used to regulate its multidrug resistance, including doxorubicin and paclitaxel [242]. As Dox kills breast cancer cells by inducing DNA damage, FOXM1 contributes to DNA repair via directly regulating DNA repair genes, which confers chemoresistance to breast cancer cells [243,244,245,246]. For instance, Nestal de Moraes G et al. discovered that overexpressed FOXM1 upregulates Survivin and XIAP (X-linked inhibitor of apoptosis protein) transcriptionally in response to Dox treatment [247]. Another study showed that the upregulation of FOXM1 is responsible for the development of acquired cisplatin resistance by promoting the repair of DNA damage [248].

For the resistance research of targeted drugs, it mainly focuses on the FOXO3 factor. Luo L et al. reported that FOXO3a controls IGF2/IGF-1R/IRS1 basic signaling in a negative feedback manner. This signaling pathway plays a crucial role in maintaining the therapeutic effects of Herceptin in HER2-positive breast cancer, and dysregulation of the FOXO3a pathway can lead to drug resistance, which is caused by IGF2/IGF-1R/IRS1 overexpression [249]. Specifically, FOXO3a regulates IGF2 and IRS1 expression by transcriptionally upregulating the expression of miR-128-3p and miR-30a-5p. The basic activity of IGF2/IGF-1R/IRS1 subsequently maintains the expression of PPP3CB, which is a subunit of the serine/threonine-protein phosphatase 2B. PPP3CB keeps FOXO3a from phosphorylation, and thus maintains the activity of FOXO3a. In Herceptin-resistant cells, the expression of PPP3CB is suppressed, leading to extensive phosphorylation of FOXO3a and the subsequent loss of its drug-resistance effect [250]. Moreover, it has been discovered that PKR-like ER Kinase (PERK) can confer breast cancer cells the phenotype of drug resistance [251,252]. FOXO3 directly targets PERK and upregulates its expression. However, in drug-resistant cells, FOXO3 is downregulated, and the expression of PERK is subsequently reduced. However, as the BCs are highly sensitive to PERK inhibition, the activity of PERK is constitutively elevated as an adaptive response. Therefore, the downregulation of FOXO3 can increase the drug resistance of breast cancer cells via downregulating the PERK level and triggering a compensatory response [253]. Song Y et al. discovered that enhancing the expression of FOXO3a by activator can reverse 5-fluorouracil resistance in breast cancer cells via arresting the G0/G1 cell cycle and promoting apoptosis [254].

## 4. Discussion

Currently, the scientific community possesses an extensive and deep comprehension of the oncogenic function of the FOX family, with the role of FOX elements in cancer gradually being clarified. As we declared in the introduction, the FOX TFs we discussed tend to act on several major pathways, such as BRCA1, HER2, TGF-β, and EGF pathways. In particular, FOXC1, FOXM1, and FOXO3 can all interact with BRCA1 and regulate the genome stability of breast cancer cells. Moreover, the overexpression or activation of HER2, as well as the activation of its downstream factors, is frequently observed in the context of breast cancer progression, particularly within the pathways regulated by FOX TFs. For instance, FOXF2 and FOXO3 both function as tumor suppressors in HER2-positive breast cancer and have limited expression levels. Downregulated FOXF2 and FOXO3 lead to highly-activated HER2 and uncontrolled proliferation. FOX TFs acting in the downstream pathway can also regulate the proliferation of breast cancer cells. For example, FOXA1, FOXA2, FOXC1, and FOXO3 can all interact with the PI3K/AKT/mTOR pathway and eventually regulate breast cancer cell proliferation [32,255,256,257]. EMT-related pathways include TGF-β/Smad, Wnt/β-Catenin, EGF, Notch, and MAPK signaling pathways. FOXC1 and FOXF2 have interactions with TGF-β/Smad, which indicates their role in breast cancer cell EMT. Another EMT pathway, Wnt/β-Catenin, is also the common mechanism for FOXC1, FOXF2, and FOXM1 to regulate breast cancer EMT [45,67,242]. This overlap phenomenon indicates that FOX family members may converge on a common signaling pathway, and an aberrantly expressed signaling pathway may be the consequence of the collective effect of various FOX TFs and other factors.

Moreover, the HER2 signaling pathway has an intricate interplay with ER and AR (androgen receptor) via the PI3K/AKT/mTOR and IGF2/IGF-1R/IRS1 pathways [147]. Specifically, HER2 can phosphorylate ER and AR and increase their activity [147]. Additionally, AR can directly target ER and increase its recruitment to ER response elements, thereby increasing the expression of ER downstream genes [258]. As we discussed previously, FOXA1 can facilitate the binding of ER and AR to their target genes, which is particularly important in luminal breast cancer [88]. In contrast, FOXO3a, which commonly functions as a tumor suppressor, can inhibit the transcriptional activity of ER and AR [254]. One study proposed the possibility of cooperation among several FOX TFs: FOXA1 first turns on chromatin in breast cancer, allowing nearby ER α binding, and then FOXM1 can replace FOXA1 and activate cell cycle genes [5,88,259]. In ER-positive breast cancer, FOXC1, FOXF2, and FOXO3 are related to the decrease in the ER level [126,258,260].

Furthermore, unlike other typical transcription factors, FOX TFs can either promote or inhibit cancer through multiple signaling pathways in different types of breast cancer, contingent on the specific cancer environment and mode of participation. Even for the same target, different FOX TFs can influence breast cancer cells in varying ways. For instance, FOXF2 plays diverting roles on WNT2B and FZD1 promoters in lumenal breast cancer and BLBC [45]. FOXF2 is minimally expressed in lumenal breast cancer and promotes the proliferation of cancer cells, while it is highly expressed in BLBC and reduces the stemness of cancer cells. However, the duality of FOXF2 is not limited to this. Even in the same breast cancer, the FOX factor also has different or even opposite effects on breast cancer due to different expression levels. Research has shown that the high expression of FOXF2 in BLBC can reduce cancer cell stemness, while the low expression of FOXF2 can promote cancer cell metastasis [48,153]. The complex nature of FOX factors makes it difficult for people to summarize the rules of their mechanisms of action, and the different effects of different expression levels of FOX in specific types of cancer will be a major challenge in subsequent research. Objectively, FOX TF plays a key role in the treatment of breast cancer, although these roles have been ignored for a long time. As we mentioned earlier, the FOX TF is associated with the drug resistance of various therapeutic drugs for breast cancer, including targeted therapy, chemotherapy, and endocrine therapy. This is also the focus of current research, that is, how to overcome the drug resistance to breast cancer cells caused by FOX TF, or how to avoid or reduce drug resistance by regulating FOX TF. Many studies have confirmed that the knockout of specific FOX TFs can affect drug resistance in some breast cancers. For example, Kumar, U. et al. showed that the knockout of FOXA1 in ER-positive breast cancer can improve the sensitivity of chemotherapy drugs (such as paclitaxel) [261]. More and more studies have shown that this is not an individual case. We believe that more studies are needed to prove the more common influence of FOX TF on drug resistance to breast cancer. In addition, FOX TF itself has the potential as a target for breast cancer treatment. Although FOX TF is rarely directly used as a drug target, it is not uncommon to study the treatment of breast cancer by acting upstream of FOX and regulating the expression of FOX. As we mentioned in the previous treatment section, Tel has been confirmed to down-regulate the expression of FOXA1 through the IGF1-R/AKT/FOXA1 pathway, to inhibit the proliferation of cancer cells in ER-positive breast cancer.

At the same time, we have also identified significant challenges that current research institutions are facing. We mentioned some drugs in the treatment section, most of which are in the preclinical stage or other drugs that have been found to have drug-hosting properties. This is a major difficulty in the clinical translation of FOX TFs, as few drugs can undergo clinical trials. This may be due to researchers’ lack of trust in the therapeutic potential of FOX TFs or a lack of successful cases. Another difficulty is the complexity of FOX TF. As we just discussed, FOX TF itself is diverse, and it has different or even opposite roles in different types of breast cancer. This complexity makes it more difficult to study FOX TF and its related breast cancer treatment.

Nevertheless, we believe that FOX TF still has considerable potential in the treatment of breast cancer. For existing therapies, resistance research based on FOX TFs can effectively address pressing issues, and combination therapy may become a more rational treatment option. In addition, many FOX TFs have been proven to have the potential to become therapeutic targets for breast cancer, which also means that it is possible to provide patients with more personalized treatment programs. They can also be used as relatively accurate biomarkers, which have considerable value for the diagnosis and prognosis of breast cancer. To sum up, this paper described the carcinogenic mechanism and regulatory pathway of some FOX TFs related to breast cancer and explained their difficulties and prospects in the treatment of breast cancer. We hope this paper can provide some enlightenment for the treatment strategy for breast cancer.

## Figures and Tables

**Table 1:** Factors relevant to FOX in breast cancer.

Relevant Factors	Fox Genes	Expression in Cancer Cells	Cell Line	Result	Reference
GATA3	FOXA1 ↑	Up	T47D and ZR75-1	Upregulated GATA3 causes overexpression of FOXA1, leading to abnormal proliferation and differentiation of cancer cells by regulating ESR1.	[26]
KMT2D	FOXA1 ↑	Up	T47D and MCF7	Inhibition of PI3K α enhances KMT2D activity and then promotes the invasion of ER+ breast cancer through the ER-FOXA1-PBX1 network.	[27]
HIF-2α	FOXA1 ↑	Up	MCF7L, T47D, and 600MPE	FOXA1 up-regulates HIF-2α in ER+ breast cancer to promote cancer cell metastasis and endocrine resistance.	[28]
TWIST1	FOXA1 ↓	Up	MCF7	Twist recruited NuRD to silence the transcription activity of the FOXA1 promoter, thereby inhibiting the expression of ER α and inducing EMT in breast cancer	[29]
	FOXA1 ↓	Up	MCF-7	Twist downregulates FoxA1 to increase the level of choline kinase in ER breast cancer and promote cancer cell metastasis.	[30]
			MCF-7, ZR-75-1, SK-BR-3, MCF 10A, MCF 12A, and MDA-MB-231	Twist upregulates miR-22 and subsequently downregulates ER α expression, promoting the ER-negative phenotype in breast cancers.	[31]
PGC-1β	FOXA2 ↓	Up	MCF-7, MCF-10A, and MDA-MB-231	Downregulation of PGC-1 β combined with overexpression of FOXA2 inhibits cancer cell migration and proliferation through the PI3K AKT mTOR signaling pathway.	[32]
CD44	FOXA2 ↑	Up	MDA-MB-231, MCF7, and ZR75	CD44 upregulates the expression of FOXA2 in the cytoplasm, thereby reducing E-Cadherin expression, promoting mesenchymal transition, and facilitating cancer cell metastasis and invasion.	[33]
FOXP2	FOXA2 ↑	Up	MCF-7, MDA-MB-231, BT-474, MDA-MB-453, BT-549, ZR-75-30, and HCC-1937, MDA-MB-436 and MDA-MB-468	FOXA2 interacts with FOXP2 to activate E-cadherin, and under the action of E-cadherin, FOXA2 downregulates ZEB2, thereby inhibiting EMT in cancer cells.	[34]
BRCA1	FOXC1 ↓	Down	HCC1937 and MDA468	BRCA1 and GATA3 synergistically inhibit FOXC1 transcription, thereby suppressing cancer cell metastasis in BLBC.	[35]
	FOXO3 ↑	Up	MCF-7, MDA-MB-231, MDA-MB-436, MDA-MB-468 and HCC70	BRCA1 can downregulate EZH2, thereby upregulating FOXO3 and inhibiting cancer cell proliferation.	[36]
EZH2	FOXC1 ↓	Up	HMEC, MCF-7, T47D, ZR751, BT474, MDA-MB-468, BT549, HS578T, and HCC1806	EZH2 inhibits FOXC1 under the action of H3K27me3, thereby enhancing cancer cell invasion in Luminal B breast cancer.	[37]
	FOXO3 ↓	Down	MDA-MB231, HCC1937 and MCF7	EZH2 can downregulate FOXO3 by recruiting NIR, thereby promoting cancer cell proliferation.	[38]
NF-κB	FOXC1 ↑	Up	MDA-MB-468, MDA-MB-231, and BT-20	EGF/EGFR can phosphorylate NF—κ B in BLBC, which then acts on the promoter of FOXC1 and upregulates its expression, thereby promoting cancer cell proliferation and migration.	[39]
LNC FOXCUT	FOXC1 ↑	Up	-	Lnc FOXCUT can directly localize upstream of the FOXC1 promoter, while the FOXCUT–FOXC1 axis can promote cancer cell metastasis through the PI3K/AKT pathway.	[40]
LNC DANCR	FOXC1 ↑	Up	-	Lnc DANCR can form a complex with EZH2, which inhibits the binding of EZH2 to FOXC1, leading to the malignant phenotype of TNBC.	[40]
LINC01123	FOXC1 ↑	Up	MCF-10A, MDA-MB-231, MDA-MB-468, MCF7 HCC1937	FOXC1 can activate LINC01123, thereby promoting the proliferation of TNBC through the miR-663a/CMIP pathway.	[41]
CXCR4	FOXC1 ↑	Up	MDA-MB-231 and BT549	FOXC1 increases the transcription of CXCR4 to promote cancer cell metastasis in TNBC.	[42]
WNT5A	FOXC1 ↑	Up	MDA-MB-231	FOXC1 binds to the WNT5A promoter and upregulates its expression, thereby activating NF—κ B and MMP expression and promoting cancer cell invasion in TNBC.	[43]
CIRCIRAK3	FOXC1 ↑	Up	MDA-MB-231	FOXC1 downregulates miR-3607 to upregulate FOXC1, thereby promoting cancer cell migration.	[44]
NCOA3	FOXF2 ↑	Up	MCF-7, T-47D, MDA-MB-231, and BT-549	FOXF2 initiates NCoA3 recruitment, thus activating the transcription of WNT2B and FZD1, and ultimately enhancing the cancer cell phenotype in luminal breast cancer.	[45]
NCOR1	FOXF2 ↑	Up	MCF-7, T-47D, MDA-MB-231, and BT-549	FOXF2 initiates NCoR1 recruitment, thereby inhibiting the transcription of WNT2B and FZD1, and ultimately suppressing cancer cell metastasis in BLBC.	[45]
CDK2	FOXF2 ↑	Down	MCF10A, HBL100, MDA-MB-468, BT549, MDA-MB-157, MDA-MB-231, and MDA-MB-435	In luminal and HER2-positive breast cancer, FOXF2 inhibits the CDK2-Rb-E2F cascade, ultimately inducing cancer cell apoptosis.	[46]
BMP4	FOXF2 ↑	Up	MDA-MB-231, 4T1, and MCF-7	In BLBC, FOXF2 activates the BMP4/Smad1 signaling pathway, leading to the promotion of cancer cell metastasis.	[47]
MIR-182-5P	FOXF2 ↓	Up	MDA-MB-231, BT-549, and MCF7	In BLBC, the TGF—β/SMAD/miR-182-5p pathway downregulates FOXF2, thereby promoting cancer cell metastasis.	[48]
YTHDF1	FOXM1 ↑	Up	MCF-10A, MCF-7, MDA-MB-231, SKBR3, T47D, and BT-549	YTHDF1 upregulates FOXM1 modified by m^6^A, thereby promoting cancer cell proliferation and invasion.	[49]
FBXL19-AS1	FOXM1 ↑	Up	MCF-10A, MCF-7, BT-549, MDA-MB-231, and SKBR3	FBXL19-AS1 downregulates miR-876-5p, thereby upregulating FOXM1 expression and promoting cancer cell proliferation.	[50]
RNF168	FOXM1 ↓	Down	MCF-7	RNF 168 downregulates FOXM1 under the action of RNF8, thereby inhibiting cancer cell proliferation.	[51]
OTUB1	FOXM1 ↑	Up	MCF-7	OTUB1 can bind and stabilize FOXM1, thereby enhancing the damage of cancer cells to DNA.	[52]
AURORA-A	FOXM1 ↑	Up	MDA-MB-231 and MCF-7	Aurora-A can prevent FOXM1 ubiquitination, thereby promoting cancer cell proliferation in TNBC.	[53]
USP39	FOXM1 ↑	Up	MCF10A, SUM185, BT474, SUM190, BT549, MDA-MB-231, MDA-MB-453, MCF-7, ZR-75-30, MDA-MB-468, SUM159, and HEK 293T	FOXM1 can upregulate USP39, thereby promoting cancer cell proliferation.	[54]
MIRNA-4521	FOXM1 ↓	Down	MCF-10A, MCF-7, and MDA-MB-468	MiRNA-4521 can downregulate FOXM1, thereby inducing cancer cell apoptosis.	[55]
MIR-671-5P	FOXM1 ↓	Down	MDA-MB-231, Hs578T, SKBR3, BT-20, MDA-MB-468, MCF-7, and T47D	MiR-671-5p can downregulate FOXM1, thereby inhibiting EMT in cancer cells.	[56]
MIR-802	FOXM1 ↓	Down	MCF-7, MDA-MB-453, MDA-MB-468, ZR-75-1, and HBL-100	MiR-802 can downregulate FOXM1, thereby inhibiting cancer cell proliferation.	[57]
ERα	FOXM1 ↑	Up	MCF-7, ZR-75-1, MDA-MB-231, and COS-1 cells	ER activates FOXM1 through ERE, thereby exerting a pro-cancer effect.	[58]
ERβ1	FOXM1 ↓	Down	CAL51, MCF-7, MCF-7(ER-), MDA-MB-231, SKBR-3, T47D, ZR-75-1, and ZR-75-1 (ER−)	ER β 1 can compete with ER α for binding to ERE, thereby inhibiting the activation of FOXM1 and exerting anticancer effects.	[59]
C-SRC	FOXM1 ↑	Up	Mammary tumors excised from female MMTV-PyMT mice	Phosphorylation of c-Src and upregulation of FOXM1 promotes cancer cell proliferation.	[60]
SEPIN-1	FOXM1 ↓	Down	BT-474, MCF7, MDA-MB-231, and MDA-MB-468	Sepin-1 downregulates FOXM1 through the Raf/Mek/Erk pathway, thereby inhibiting cancer cell proliferation.	[61]
CYCLIND-CDK4/6	FOXM1 ↑	Up	-	CyclinD-CDK4/6 can phosphorylate FOXM1 to maintain stability, thereby exerting a pro-cancer effect.	[62]
CYCLINE/A-CDK2	FOXM1 ↑	Up	-	CyclinE/A-CDK2 can upregulate FOXM1 transcription, thereby exerting a pro-cancer effect.	[63,64]
CYCLINB-CDK1	FOXM1 ↑	Up	-	CyclinB-CK1 can upregulate FOXM1 transcription, thereby exerting a pro-cancer effect.	[63,64]
HMGA1	FOXM1 ↑	Up	MDA-MB-231, MDA-MB-157, and HEK293T	HMGA1 can upregulate FOXM1 to promote the angiogenesis process of TNBC.	[65]
SLUG	FOXM1 ↑	Up	MCF-7 and MDA-MB-231	FOXM1 can upregulate Slug to inhibit E-cadherin, thereby promoting cancer cell migration.	[66]
KIF23	FOXM1 ↑	Up	MCF-7, ZR-75-1, BT474, MDA-MB-231, MDA-MB-468, HCC1806, and MCF10A	FOXM1 can upregulate KIF23 to activate the Wnt/β—catenin pathway, thereby promoting EMT in cancer cells in TNBC.	[67]
INTEGRIN β1	FOXM1 ↑	Up	BT-20, MDA-MB-231, MDA-MB-453, MDA-MB-361, and ER+ MCF7	Integrin β 1 can bind to FOXM1 to upregulate eEF-2K, thereby promoting cancer cell proliferation and invasion in TNBC.	[68]
CRY2	FOXM1 ↑	Down	MCF-10A, MCF-7, T47D, BT474, MDA-MB-231, and BT549	FOXM1/DNMT3b complex formed by FOXM1 can bind and down-regulate the expression of CRY2, thus increasing the aggressiveness of cancer cells in ER+ breast cancer.	[69]
FZD5	FOXM1 ↑	Up	MDA-MB-231, MDA MB-468, and Hs-578t	FZD5 can upregulate FOXM1 expression through the Wnt/β—catenin pathway, thereby upregulating BRCA1 and BIRC5 and ultimately promoting cancer cell proliferation.	[70]
CBP	FOXM1 ↑	Up	MDA-MB-231, MDA-MB-468, MDA-MB-436, and Hs578T	FOXM1 can bind to CBP/β—catenin to form a complex and promote the increase in CSC.	[71]
CDH	FOXM1 ↑	Up	-	FOXM1 can upregulate CDH and promote cancer cell stemness in TNBC.	[72]
YAP	FOXM1 ↑	Up	MCF-7, MDA-MB-231, SK-BR-3, and MCF-10A	FOXM1 can upregulate YAP to promote cancer cell proliferation and migration.	[73]
AURKA	FOXM1 ↑	Up	MDA-MB-231, SUM-149, MCF-7, and MCF-10 A	FOXM1 can upregulate AURKA to promote cancer cell proliferation and metastasis.	[74]
BECLIN-1 LC-3	FOXM1 ↑	Up	MDA-MB-231, BT-549 ER+ MCF-7, and MCF10A	FOXM1 can upregulate Beclin-1 and LC-3 to enhance the autophagy process	[75]
MIR-186-5P	FOXM1 ↑	Up	MDA-MB-231, BT-549, MCF-7, and MCF10A	FOXM1 upregulates miR-186-5p, thereby promoting Beclin expression and promote cancer progression	[76]
MIR-200B-5P	FOXM1 ↑	Down	MDA-MB-231, BT-549, MCF-7, and MCF10A	FOXM1 downregulates miR-20b-5p, which promotes cancer cell proliferation	[76]
CIRC-FOXO3	FOXO3 ↑	Down	-	Circ-Foxo3 binds to MDM2 to upregulate FOXO3, thereby inducing cancer cell apoptosis.	[77]
EP300	FOXO3 ↑	Down	BT474, MCF-7	EP300 acetylates the FOXO3 gene and boosts its cancer-suppressive effects.	[78]
GEMININ	FOXO3 ↓	Up	HEK-293T, MCF-7, MDA–MB-231, HCT116, MCF-10A, and 3T3-L1	Geminin downregulates FOXO3 transcriptional activity through HDAC3, thereby downregulating Dicer and ultimately promoting cancer cell metastasis.	[79]
FLOTILLIN-2	FOXO3 ↓	Up	MCF-7 and MDA-MB-231	Flotilin-2 can downregulate FOXO3 through the PI3K/AKT pathway, thereby promoting cancer cell proliferation.	[80]
MIR-940	FOXO3 ↓	Up	T47D and MCF-7	MiR-940 can directly downregulate FOXO3, thereby promoting the malignant phenotype of cancer cells.	[81]
SOX2	FOXO3 ↓	Up	MCF-10A, MCF-7, T47D, MDA-MB-231, BT549, BT474, SKBR3, and Hs578T	SOX2 can activate DNMT1 to downregulate FOXO3, leading to malignant progression of cancer cells.	[82]
BMF	FOXO3 ↑	Down	KP8 and KEP1 derived from tumors that developed in female mice	FOXO3 can activate BMF, thereby promoting cancer cell apoptosis.	[83]
MIR-29B-2	FOXO3 ↑	Down	MCF-7, BT474, BT549, MDA-MB-231, Hs578T, HCC38, and HCC1937	FOXO3 can upregulate miR-29b-2 to downregulate VEGF-A, thereby inhibiting cancer metastasis and invasion.	[84]
MIR-338	FOXO3 ↑	Down	MCF-7, BT474, BT549, MDA-MB-231, Hs578T, HCC38, and HCC1937	FOXO3 can upregulate miR-338 to downregulate NRP1, thereby inhibiting cancer metastasis and invasion.	[84]

**↑:** UPREGULATION; ↓: Downregulation.

**Table 2:** FOX-related drugs with therapeutic effect on breast cancer.

Drug Name	Cancer Subtype	Cell Model	Related Fox TF	Clinical Progress	Reference
TELAPREVIR	ERα+ breast cancer	MDA-MB-231, MCF-7, T47D-1, BT-474, SKBR3, and AU565 cells	FOXA1	Preclinical stage	[222]
PYROTINIB	HER2+ breast cancer	SK-BR-3 and AU565 cells	FOXC1	Phase III clinical trials	[223]
N-3	TNBC	MDA-MB-231 and 4T1 cells	FOXC1	Preclinical stage	[224]
NB-55	TNBC	MCF-7, T47D, MDA-MB-231 cells	FOXM1	Preclinical stage	[225]
CRBN-RECRUITING MOLECULE	TNBC	MDA-MB-231 cell	FOXM1	Preclinical stage	[226]
DISULFIRAM	No specific type specified	MCF-7, MDA-MB-231, and HBT-3 cells	FOXO3	Preclinical stage	[227]
